# The Role of TGF-β Signaling Pathway in Determining Small Ruminant Litter Size

**DOI:** 10.3390/biology14070786

**Published:** 2025-06-29

**Authors:** Ying Han, Guiling Cao, Wenting Chen, Changfa Wang, Muhammad Zahoor Khan

**Affiliations:** College of Agriculture and Biology, Liaocheng University, Liaocheng 252059, China

**Keywords:** sheep, goat, litter size, *GDF9*, *BMPs*, *Inhibin*, *SMADs*, *AMH*

## Abstract

The transforming growth factor-beta (TGF-β) superfamily plays a crucial role in regulating female reproductive traits, particularly litter size, in small ruminants, such as sheep and goats. This review comprehensively examines the molecular mechanisms through which TGF-β superfamily members—including bone morphogenetic proteins (*BMPs*), growth differentiation factor 9 (*GDF9*), inhibin (*INHA* and *INHB*), and associated signaling genes—influence ovarian follicular development, ovulation rate, and ultimately, litter size. We synthesize recent findings on polymorphisms in key genes, such as *BMPR1B, BMP15*, *GDF9*, *inhibins* and *SMADs* family genes, across diverse sheep and goat breeds worldwide. The manuscript highlights how specific mutations in these genes create an intricate signaling network that modulates granulosa cell proliferation, follicular sensitivity to FSH, and the prevention of dominant follicle selection. These molecular interactions result in increased ovulation rates and larger litter sizes in prolific breeds. The gene dosage effects observed in heterozygous versus homozygous mutation carriers further illuminate the complex nature of these reproductive regulations. This improved the understanding of the genetic basis for prolificacy provides valuable insights for marker-assisted selection strategies aimed at enhancing reproductive efficiency in small ruminant breeding programs, with significant implications for improving livestock productivity and economic outcomes.

## Simple Summary

This review investigates how specific genes control the number of offspring that sheep and goats produce per pregnancy, which is crucial for farm profitability. We examine key members of the transforming growth factor-beta (TGF-β) superfamily, including BMP15, GDF9, BMPR1B, and associated genes, to understand how they influence litter size across different breeds worldwide. An analysis of published literature demonstrates that natural mutations in these genes create a complex communication network between oocytes and the surrounding granulosa cells, ultimately determining follicular development and ovulation rate. When these genes have certain mutations, they alter the ovary’s sensitivity to reproductive hormones, allowing multiple follicles to mature instead of just one or two, resulting in larger litters. Furthermore, the research revealed that having one copy of a beneficial mutation often increases fertility, while having two copies can sometimes cause infertility, showing the delicate balance required for optimal reproduction. These findings provide valuable genetic markers that farmers and breeders can use to select animals with higher reproductive potential, leading to increased livestock productivity and improved economic outcomes. This knowledge offers practical tools for enhancing breeding programs, ultimately contributing to more efficient and sustainable sheep and goat production systems that can better meet global food security needs.

## 1. Introduction

Reproductive efficiency, particularly litter size, represents a critical economic trait in small ruminant production systems worldwide [1,2,3]. The number of offspring produced per pregnancy directly influences overall productivity and profitability in sheep and goat farming operations. Over the past several decades, significant advances in reproductive genetics have illuminated the molecular mechanisms underlying prolificacy in these species, with the transforming growth factor-beta (TGF-β) superfamily emerging as a central player in this complex biological process [4].

The TGF-β superfamily encompasses a diverse group of structurally related but functionally distinct growth factors that regulate numerous physiological processes, including cellular proliferation, differentiation, migration, and apoptosis [5]. Within this superfamily, bone morphogenetic proteins (*BMPs*), growth differentiation factor 9 (*GDF9*), inhibins (*INHA* and *INHB*), and their associated signaling molecules have been particularly implicated in female reproductive function [2]. These genes establish an elaborate communication network between the oocyte and surrounding somatic cells that ultimately determines follicular development trajectories and ovulation outcomes.

Recent research has identified naturally occurring polymorphisms in TGF-β superfamily genes that significantly affect litter size across various sheep and goat breeds. These genetic variants modify signaling pathways in ways that alter follicular development dynamics, follicle-stimulating hormone (FSH) sensitivity, and the selection mechanisms that typically limit the number of follicles reaching ovulation [6]. Among these, mutations in *BMPR1B* (also known as FecB or the Booroola gene), *BMP15* (FecX), *GDF9* (FecG), mothers against decapentaplegic (*SMAD*) family genes, *INHA*, and *INHB* have demonstrated the most pronounced effects on ovulation rate and litter size, with certain combinations of mutations exhibiting additive or even multiplicative influences on prolificacy [7,8,9,10].

This review aims to synthesize current knowledge regarding the roles of TGF-β superfamily members in regulating litter size in small ruminants. We highlighted the key genes of the TGF-β signaling pathway and their polymorphisms association with female reproductive efficiency with specific focus on litter size in sheep and goats. Furthermore, we discussed the molecular crosstalk between oocyte-derived factors and granulosa cells, evaluate how specific genetic variants disrupt normal signaling dynamics, and explore the downstream consequences for follicular development and ovulation. By integrating findings from molecular genetics, reproductive physiology, and breeding studies, we provide a comprehensive framework for understanding the genetic architecture underlying prolificacy in sheep and goats. This knowledge not only advances our fundamental understanding of reproductive biology but also offers practical applications for marker-assisted selection programs aimed at enhancing reproductive efficiency in small ruminant production systems.

## 2. Methodology for Literature Search

A comprehensive literature search was conducted to investigate the role of TGF-β signaling pathway and its members in reproductive efficiency, with particular emphasis on litter size in sheep and goats. The search strategy employed multiple electronic databases including Google Scholar, Web of Science, X-MOL, and PubMed. Primary keywords included “litter size”, “reproductive efficiency”, “small ruminants”, “sheep”, “goats”, “genetic polymorphisms”, “TGF-β signaling pathway”, along with specific gene identifiers, including “*BMPs*”, “*INHA*”, “*INHB*”, “*GDF9*”, anti-müllerian hormone (*AMH*) and “*SMADs*.”

The temporal scope of the search covered articles published from 2018 through April 2025, though selected seminal studies published as early as 2007 were incorporated to provide essential background context and historical perspective. Inclusion criteria required all articles to be indexed in Science Citation Index (SCI) journals and published in the English language. Study exclusion criteria eliminated non-SCI indexed publications, articles in languages other than English, book chapters, conference abstracts, and unpublished research data.

## 3. The TGF-β Superfamily Genes Role in Sheep and Goats Litter Size

The association of TGF-β Superfamily members have been well with litter size in sheep and goats [4]. Transforming growth factor-β1 (TGF-β1) is a multifunctional growth factor that is crucial in regulating various physiological processes, including embryonic growth and development [5]. These signaling pathways modulate follicular development, ovulation rates, and ultimately, fecundity in these economically important livestock species. TGIF1, which is also known as TGF-β induced factor homeobox 1, has been found to help change how sensitive FSH-β is to pulses of gonadotropin-releasing hormone (GnRH), which is critical for follicular development and ovulation [1]. This homeodomain protein serves as a transcriptional repressor and modulates TGF-β signaling, providing a molecular link between this pathway and reproductive outcomes.

Multiple studies have identified significant associations between genetic variants in TGF-β superfamily genes and reproductive performance. Consistently, a study reported through association analysis that a synonymous mutation at g.37871539C>T in *TGIF1* was highly associated with litter size in small-tailed Han sheep [11]. Furthermore, given the association of the *TGIF1* g.37866222C>T polymorphism with litter size in small-tailed Han sheep (*p* < 0.05), fecundity differences might be caused by the change in amino acid from proline (Pro) to serine (Ser), which has an impact on secondary, tertiary protein structures with concomitant TGIF1 functionality changes [12]. A study reported that four SNPs (g.9414A>G, g.28881A>G, g.28809T>C, g.10429G>A) in *TGFβRI* and one *TGFβRII* SNP (g.63940C>T) were significantly associated with litter size in Tibetan sheep [13]. Recent investigations have elucidated specific molecular pathways through which TGF-β family members influence ovarian function. Consistently, they revealed that *TGF-β1* mediates the novel-m0297-5p targeting of *WNT5A* to inhibit granulosa cell proliferation and activity in small-tailed Han sheep, providing insights into the regulatory mechanisms of follicle development [5].

In line studies on genetic basis of high fecundity in Hu sheep found that all experimental animals were homozygous for the *BMPRIB* (A746G) mutation, with significant differences in BMP/Smad pathway gene expression between high-fecundity and low-fecundity sheep groups [8,10]. Specifically, *BMP4*, *BMPRIB*, *BMPRII*, *SMAD4*, *GDF9*, and *TGF-βRI* mRNAs were more abundant in high-fecundity animals, while *BMP15* mRNA was less abundant, suggesting that unidentified genetic factors may influence ovulation rate through this pathway [10]. The accumulated evidence demonstrates that TGF-β superfamily members and their signaling components play integral roles in determining litter size in small ruminants. Genetic variants within these pathways represent promising markers for selective breeding programs aimed at enhancing reproductive performance in sheep and goat populations.

### 3.1. Association of BMP Family Genes and GDF9 with Litter Size in Goats

Members of the transforming growth factor-beta (TGF-β) superfamily, particularly the bone morphogenetic proteins (*BMPs*) family genes and growth and *GDF9*, serve critical functions in ovarian follicular development, cellular differentiation, cumulus expansion, and ovulation regulation [6,14,15]. The *BMP15* and *GDF9* operate as oocyte-secreted factors that exert significant regulatory control over female reproductive processes, influencing both somatic granulosa cell fate determination and oocyte developmental competence [15,16,17,18]. Recent investigations have demonstrated that *GDF9* and BMP receptors (*BMPRs*) enhance proliferation in both granulosa and theca cell populations, further supporting their role in folliculogenesis [19]. Extensive research has established the fundamental role of *BMP* family genes in caprine litter size determination. Significant associations have been documented for several key genes. Multiple studies have confirmed *BMP4*’s involvement in regulating reproductive parameters in goats [15,20]. The association between *BMP15* polymorphisms and reproductive traits has been thoroughly investigated across diverse caprine populations [9,21,22,23,24,25]. The *BMPR1B* gene, also designated as *FECB*, has demonstrated consistent associations with reproductive efficiency in goats [26,27,28,29,30].

Significant lambing rate differentials have been observed in relation to specific genotypic variations. Individuals exhibiting CC and CT genotypes at the FecB C94T locus demonstrated substantially higher lambing rates compared to TT genotype carriers, with increases of 45.7% and 46.8%, respectively. Additionally, individuals with the CC genotype at the ESR C463T locus exhibited significantly elevated lambing rates relative to both CT and TT genotypes, with increases of 9% and 15%, respectively [27]. Consistently, Wang Y et al. [25] employed polymerase chain reaction–single-strand conformation polymorphism (PCR-SSCP) analysis and DNA sequencing to examine exon 2 of the *BMP15* gene in indigenous Chinese goat breeds. Their findings revealed that Funiu white goats possessing the BB genotype exhibited significantly enhanced litter size at birth, averaging 0.91 or 0.82 more offspring compared to AB or AA genotype carriers, respectively. Furthermore, they emphasized that second-parity litter size constitutes a critical indicator of caprine prolificacy, as it demonstrates the capacity for consistent multiple offspring production across consecutive pregnancies—a key parameter of reproductive fitness [25]. Functional polymorphisms in *GNRH1* (g.3548A>G and g.3699G>A) and *GDF9* (g.4093G>A) demonstrate significant associations with litter size variation, suggesting their utility as molecular markers for marker-assisted selection in goat breeding programs [30]. Consistent with these observations, additional research has identified associations between specific SNPs (g.3905A>C and g.4135G>A) and litter size in Shaanbei white cashmere goats [31]. A substantial body of evidence has consistently demonstrated associations between GDF9 polymorphic variants and litter size across diverse goat breeds [23,32,33,34,35]. The collective evidence strongly supports that *BMP* family genes and *GDF9* exhibit robust associations with caprine litter size, attributable to their fundamental roles in folliculogenesis, ovulatory regulation, and the modulation of follicular responsiveness to gonadotropic signaling pathways. These molecular genetic insights highlight the potential application of these genes as selection markers in precision breeding programs aimed at enhancing reproductive efficiency in goat populations.

### 3.2. Role of BMP Family Genes and GDF9 in Litter Size in Sheep

Litter size, a critical reproductive trait in sheep breeding, is influenced by genetic factors, such as *BMP15*, *GDF9*, and *BMPR1B*, which regulate ovarian follicle development and ovulation. Mutations in *BMP15* (e.g., c.31_33CTTinsdel in Hu sheep) and *GDF9* (e.g., S395F/S427R in Chinese breeds) enhance litter size by disrupting granulosa cell function or oocyte maturation [36,37,38], while *BMPR1B* polymorphisms, like c.746A>G, improve fecundity in Ujimqin and crossbred sheep [39]. Geographically, studies from China dominate, with *BMP15* variants (e.g., g.50985975G>A in Mongolian sheep) and *BMPR1B* mutations (e.g., g.29362047T>C in Hu sheep) being prominent [40,41], though international research confirms these genes’ roles in Turkish Akkaraman (also known as white Karaman) and Australian white sheep [42,43], suggesting conserved mechanisms with breed-specific allele frequencies. Polygenic interactions, such as synergies between *BMPR1B*, *BMP15*, and *GDF9* in Han and Hu sheep [44] or *BMPR1B-BMP2* in Chinese indigenous breeds [45], highlight the need for multi-locus approaches in breeding. Methodologically, diverse polymorphisms—including SNPs (*BMPR1B* c.746A>G), indels (g.30058882_30058873GCAGATTAAA in Gobi sheep), and missense mutations (p.Q249R)—disrupt gene function [46], though reference-year inconsistencies, such as those found in Zhang et al. [36], warrant further verification. Marker-assisted selection (MAS) targeting *BMP15/GDF9* in Hu sheep [47] and crossbreeding strategies (e.g., Suffolk × Ujimqin hybrids) can optimize productivity [39]. Future research should address gene–environment interactions, epigenetics, and underrepresented breeds, like Sudanese desert sheep [48], while functional studies are needed to clarify polymorphism mechanisms. In conclusion, *BMP15*, *GDF9*, and *BMPR1B* are pivotal for litter size regulation; validating these markers across breeds and advancing genomic tools will enhance breeding efficacy and sustainable sheep production. In addition, a study investigated the C864T polymorphism in exon-9 of the *BMPR-1B* gene and its association with litter size across 596 ewes from three breeds (Dorset, Mongolian, and small-tailed Han) [49]. DNA analysis revealed two genotypes (AA and AB), with AA being most frequent. Furthermore, they reported that *BMPR-1B*-C864T mutation significantly affected litter size traits, with heterozygosity in exon-9 increasing litter size across all studied breeds [49]. To investigate the role of *BMPR1B*, *BMP15*, and *GDF9* in sheep prolificacy, ovarian tissue in polytocous small-tailed Han (STH) and monotocous Sunite (SNT) ewes showed the highest mRNA levels for all three genes, with *BMPR1B* and *GDF9* expression significantly elevated in STH ovaries and *BMP15* markedly reduced in STH pituitary, ovarian, oviduct, and uterine tissues [50]. Furthermore, they suggest *BMPR1B* and *GDF9* promote litter size through ovarian activity in the hypothalamic–pituitary–gonadal axis, while high *BMP15* expression correlates with reduced prolificacy [50]. Consistently, a study reported that GG and AA genotypes of *GDF9* gene showed reduced prolificacy in Russian sheep breeds (Salsk and Volgograd sheep) [51]. Low polymorphism was observed, with high frequencies of GG and AA homozygous genotypes, yet heterozygous AG genotypes at both sites were linked to maximal fertility [51]. Another study reported that *GDF9* and *BMPR1B* were linked to litter size in Egyptian Rahmani and Ossimi sheep rams [52]. Furthermore, it was revealed that *GDF9* and *BMP15* were exclusively expressed in oocytes, with reduced levels in poor-quality oocytes, underscoring their role in ovulation. In addition, *BMPR1B* and *BMP6* transcripts were detected in oocytes, granulosa, cumulus cells, and corpora lutea, suggesting *BMP6* may regulate follicular maturation and luteolysis via *BMPR1B* [52]. A T755C mutation in BMP15 (L252P substitution) was linked to litter size in Iranian sheep, with heterozygous (CT) ewes producing 0.24–0.30 more lambs than CC and TT genotypes [53]. Furthermore, triplet-birth and sterile ewes exclusively carried the CT genotype, suggesting dual impacts on fertility and prolificacy Iranian Afshari, Ghezel, and Shal breeds [53]. The summary of determinant genes (*BMPs* and *GDF9*) and their polymorphisms associated with litter size in sheep is provided in Table 1.

### 3.3. Effect of Inhibins, SMAD Family Genes, and AMH on Litter Size in Goats and Sheep

The transforming growth factor-beta (TGF-β) signaling pathway encompasses several key gene families that play crucial roles in reproductive physiology, cellular differentiation, and tissue growth. These include the *AMH*, inhibins, and *SMAD* family genes (Table 2), which have emerged as targets of interest for genetic improvement in breeding programs aimed at enhancing reproductive output in small ruminants [75,76].

#### 3.3.1. The *AMH* and Reproductive Performance

The *AMH*, a member of the TGF-β superfamily, has demonstrated significant associations with reproductive traits in goats. Genetic polymorphism studies have revealed critical associations between specific *AMH* gene variants and reproductive performance in goats. The SNP-g.89172108A>C polymorphism within the *AMH* gene has been particularly well-characterized, demonstrating significant correlations with litter size in both Dazu black and Chuanzhong black goat breeds [75,77]. Genotype–phenotype analyses have revealed distinct patterns of reproductive performance, with homozygous CC genotypes consistently exhibiting superior litter sizes compared to heterozygous AC genotypes, suggesting this mutation confers a reproductive advantage [77]. Similarly, in Chuanzhong black goats, animals carrying the TT genotype displayed enhanced litter sizes relative to TG and GG genotype carriers [75].

The relationship between *AMH* and reproductive traits extends beyond genetic polymorphisms to encompass serum hormone concentrations. Multiple studies have established positive correlations between serum AMH levels and various reproductive parameters, including ovarian reserve capacity, antral follicle count, ovarian surface area, ovulation rate, and ultimately, litter size [78,79]. These findings suggest that *AMH* serves as a reliable predictor of reproductive potential in goats, with higher concentrations indicating greater ovarian activity and fertility. The significance of *AMH* in small ruminant reproduction is further supported by ovine studies, where elevated AMH concentrations have been associated with increased litter sizes in Romanov sheep [80]. This cross-species consistency reinforces the fundamental role of *AMH* in regulating reproductive processes across related livestock species.

#### 3.3.2. Role of SMAD Family Genes in Reproductive Regulation

The *SMAD* family genes, comprising *SMAD1*, *SMAD2*, *SMAD3*, *SMAD4*, and *SMAD5* genes, functions as intracellular signaling mediators within the TGF-β superfamily. These proteins display widespread expression across both developmental and adult tissues, with pronounced roles in normal embryogenesis and reproductive function [8,81,82,83,84,85]. The *SMAD* family of genes plays a pivotal role in reproductive traits across various livestock species, with mounting evidence demonstrating their fundamental involvement in litter size determination. Genome-wide association studies have established *SMAD1* as a significant genetic determinant of litter size in sheep, where high-prolificacy individuals display elevated expression of this gene [86]. The molecular basis for this association stems from *SMAD1*’s activation of the BMP intracellular signal transduction pathway, which has been causally linked to accelerated ovulation in ewes [8,86,87]. This pathway represents a critical regulatory mechanism, as SMAD proteins encoded by *SMAD1* amplify the actions of key reproductive candidate genes through BMP signaling [87]. The functional significance of SMAD signaling extends beyond *SMAD1*, with the Booroola (FecB) gene demonstrating effects on progesterone levels and the expression of both *BMP* and *SMAD* signaling genes within ovine ovaries [87]. At the tissue level, *SMAD1* expression has been documented in ovarian tissues, where it contributes to the regulation of follicular growth and ovulation through multiple integrated pathways, including estrogen, TGF-β, retrograde endocannabinoid signaling, and the Hippo pathway [87,88]. Comprehensive expression analysis in Tibetan sheep has revealed widespread tissue distribution of *SMAD1*, *SMAD2*, and *SMAD3* genes, with particularly elevated expression observed in reproductive tissues, such as the uterus and ovary, as well as in the spleen and lung [89]. The reproductive relevance of SMAD signaling is further supported by evidence from multiple species. In mice, SMAD signaling within granulosa cells has been implicated in regulating metastatic behaviors and other ovarian functions [90]. Similarly, *SMAD2* exhibits a significant association with litter size and has been documented in goat ovarian tissue, underscoring its reproductive significance [90]. The functional impact of *SMAD* gene polymorphisms is evidenced by genotype–phenotype relationships; the CC genotype at the *SMAD1* g.10729C>T locus yields significantly higher litter sizes compared to the CT genotype (*p* < 0.05), while at the *SMAD3* g.21447C>T locus, the TT genotype demonstrates superior litter sizes relative to both CC and CT genotypes [7]. In goats, the collective evidence consistently demonstrates associations between the expression and polymorphisms of multiple SMAD family members (*SMAD1*, *SMAD2*, *SMAD3*, and *SMAD6*) and litter size, revealing a critical genetic component underlying reproductive trait variation [91,92,93,94]. This pattern is reinforced by additional studies identifying key genes, including *SMAD2* and *AMHR2* as contributors to litter size determination in goats [95], collectively establishing the SMAD family as a central molecular framework governing reproductive success across livestock species.

#### 3.3.3. Inhibins’ Role in Follicular Development and Litter Size

The reproductive physiology of animals is significantly influenced by inhibins, glycoproteins predominantly synthesized by ovarian granulosa cells that serve as negative feedback regulators of FSH secretion in the anterior pituitary [96,97]. This regulatory mechanism forms a critical component of the hypothalamic–pituitary–gonadal axis, orchestrating follicular development and ovulation events [98]. The *INHA* gene specifically encodes a protein that functions as a biomarker for fully developed ovarian follicles, while simultaneously mediating FSH secretion and ovulation frequency [2,98,99]. The modulatory effect of inhibin on FSH concentrations directly impacts ovulation rates and follicular recruitment patterns [99], phenomena that have been established as key determinants of litter size in ruminant species [98]. Consistently, molecular investigations have demonstrated that genetic polymorphisms within inhibin genes correlate with reproductive phenotypes, particularly enhanced litter size outcomes in high-fertility goat populations [100,101,102,103,104,105,106]. The significance of these genetic variations is exemplified by findings at the g.28317663A>C locus of the *INHA* gene, where individuals carrying the AC genotype exhibited significantly larger litter sizes compared to those with the AA genotype [101]. This amino acid-altering SNP appears to influence protein functionality through structural modifications, establishing a direct mechanistic link between genetic variation and reproductive output. Similarly, Isa et al. documented that the CT genotype at the g.3234C>T locus in the *INHA* gene was associated with superior litter size performance relative to the CC genotype in Kalahari red and Nigerian goats [103].

The molecular framework governing these reproductive traits extends beyond inhibin genes alone, encompassing multiple components of the TGF-β signaling cascade. Comprehensive genomic analyses have identified *SMAD1* and *INHB* as additional contributors to litter size variation, with these genes influencing hormone secretion patterns (FSH and LH), placental development, embryonic viability, folliculogenesis, ovulation dynamics, and preovulatory follicle maturation in diverse sheep breeds [86]. The regulatory complexity of this system is further highlighted by the recent identification of miR-134-3p as a post-transcriptional regulator of *INHBA* in ovine granulosa cells [107]. This microRNA exhibits an inverse relationship with follicular development, decreasing in expression as follicular diameter increases. Functionally, miR-134-3p overexpression inhibits granulosa cell proliferation while promoting apoptosis, effects that are reversed upon microRNA knockdown. The mechanistic pathway involves the modulation of cell cycle progression and the suppression of the PI3K/AKT/mTOR signaling cascade, while knockdown activates this pathway [107]. The direct targeting of *INHBA* by miR-134-3p has been confirmed through co-transfection studies, demonstrating that this microRNA regulates ovine granulosa cell function via the TGF-β/PI3K/AKT/mTOR pathway [107]. This study provides interesting findings; however, future research investigating polymorphisms regulated by miR-134-3p and their effects on reproductive performance would be valuable.

**Figure 1:**
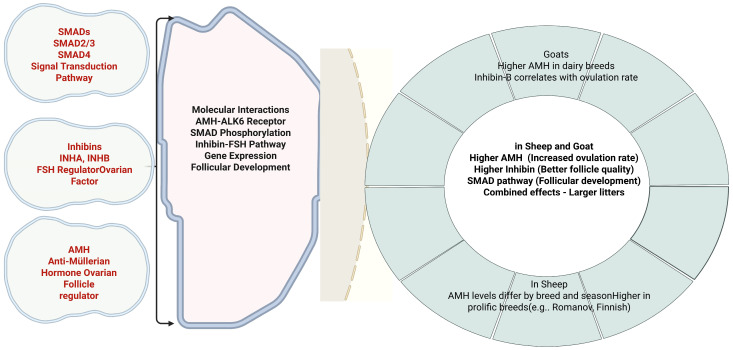
Effects of *AMH*, *SMADs*, and *Inhibins* on litter size in sheep and goats.

Additional evidence supporting the clinical relevance of inhibin genetic variants derives from studies in thin-tailed sheep, where the g.236311367G>A polymorphism in *INHA* was associated with significantly enhanced litter size in GA genotype carriers compared to individuals with homozygous AA or GG genotypes [108]. These findings collectively establish the TGF-β signaling pathway genes, particularly *AMH*, *SMAD* family members, and *inhibins*, as fundamental regulators of reproductive traits in small ruminants (Figure 1). The consistent association between specific polymorphisms and enhanced litter size performance provides a foundation for genetic marker development in selection programs targeting reproductive efficiency improvements in goat and sheep production systems. Understanding the molecular mechanisms underlying these genetic associations will enable more precise and targeted approaches to genetic improvement strategies for small ruminant production systems.

**Table 2:** *AMH*, *Inhibins,* and *SMADs* family genes’ association with litter size in goats and sheep.

Gene	SNP/Variant	Effect	Breed	Country	References
*SMAD1*	g.10729C>T	CC genotype produced significantly higher litter sizes than CT genotype	Tibetan sheep	China	[7]
*SMAD3*	g.21447C>T	TT genotype showed higher litter sizes than CC and CT genotypes	Tibetan sheep	China	[7]
*SMAD1*	rs406357666	Higher expression in high-prolificacy sheep	Hu sheep	China	[10]
*AMH*	g.89172108T>G	TT genotype exhibited higher litter size compared to TG and GG genotypes	Chuanzhong black goats	China	[75]
*AMH*	g.89172108A>C	CC genotype associated with higher litter size compared to AC genotype	Dazu black goats	China	[77]
*INHB*	rs412280524rs429836421	Associated with variation in litter size	Icelandic and Finn sheep	China	[86]
*SMAD1*		Activates BMP signaling pathway associated with accelerated ovulation	Malpura sheep	India	[87]
*SMAD2* and *SMAD1*		Associated with litter size	Shaanbei white cashmere	China	[91,92,93]
*INHA*	g.28317663A>C	AC genotype associated with significantly higher litter size than AA genotype	Hainan black goats	China	[101]
*INHA*	g.3234C>T	CT genotype associated with significantly larger litter size compared to CC genotype	West African dwarf goats	Nigeria	[103]
*INHA*		Target of miR-134-3p regulation affecting follicular development through TGF- TGF-β/PI3K/AKT/mTOR pathway in GCs	Sheep granulosa cells (GCs)	China	[107]
*INHA*	g.236311367G>A	GA genotype had significantly higher litter size than AA or GG genotype	Thin-tailed sheep	Indonesia	[108]

## 4. Discussion

**Figure 2:**
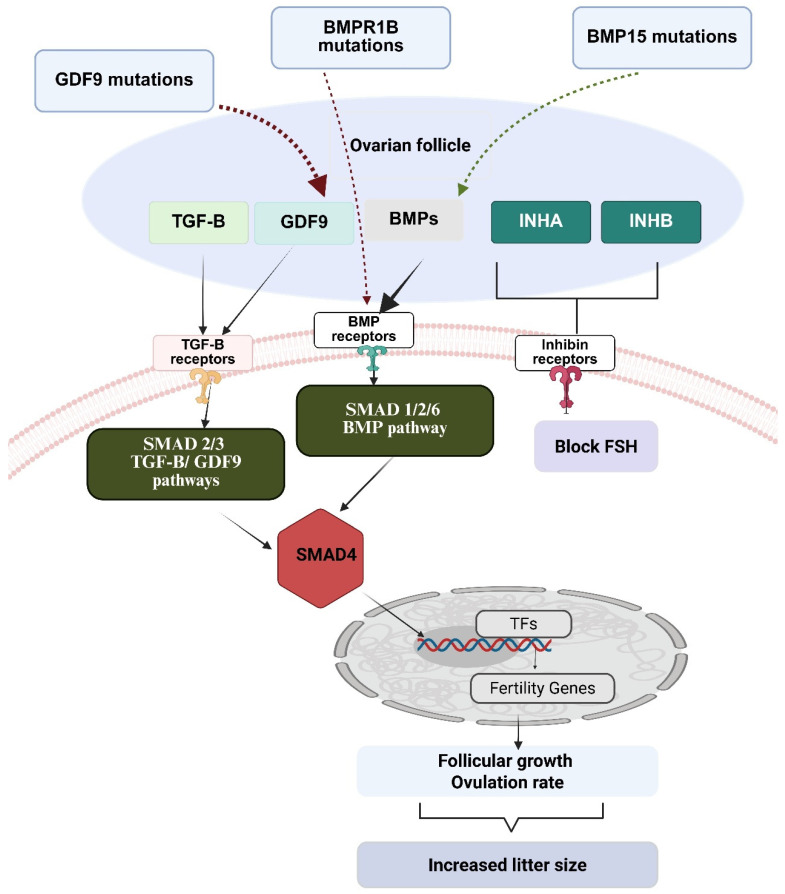
Crosstalk between TGF-β signaling members and their association with litter size in sheep and goats. This figure depicts signaling pathways in ovarian follicles mediated by TGF-β superfamily members. *GDF9*, *BMPs*, and *inhibins* activate distinct SMAD-dependent pathways that regulate fertility genes and control follicular development. Mutations in G*DF9*, *BMPR1B*, and *BMP15* alter these pathways, affecting ovulation rates and litter size. Inhibins modulate the process by blocking FSH action. These integrated pathways collectively determine follicular growth dynamics and reproductive outcomes. Note: “TFs” stands for transcription factors.

The molecular crosstalk between *BMPR1B*, *BMP15*, and *GDF9* orchestrates the ovulation rate and litter size in sheep and goats through an intricate signaling network. In this network, oocyte-derived *BMP15* and *GDF9* form heterodimers that bind to *BMPR1B* receptors on granulosa cells, activating *SMAD*-dependent pathways that regulate *FSH* sensitivity, steroidogenesis, and cell proliferation (Figure 2) [109,110,111,112]. Mutations in these genes (such as the *FecB* mutation in *BMPR1B*, various *FecX* mutations in *BMP15*, and *FecG* mutations in *GDF9*) disrupt this signaling balance by either enhancing receptor sensitivity (*BMPR1B* mutations) or altering ligand bioavailability and function (*BMP15*/*GDF9* mutations). These disruptions result in reduced negative feedback on *FSH*, accelerated follicular development, increased the recruitment of secondary follicles, and prevented dominant follicle selection. Ultimately, these changes lead to higher ovulation rates and larger litter sizes and birth weight [113]. The phenotypic expression is dependent on gene dosage effects where heterozygous mutations often increase fertility, while homozygous mutations can sometimes cause infertility due to impaired follicular development.

The regulation of litter size in small ruminants, such as sheep and goats, involves an intricate molecular crosstalk centered around the *TGF-β* superfamily signaling network. This reproductive control system begins with the oocyte, which serves as the command center for follicular development through its secretion of two critical growth factors—*BMP15* and *GDF9*. These oocyte-derived proteins form both homodimers and heterodimers, with the heterodimeric complex known as “cumulin” exhibiting significantly higher bioactivity. *BMP15* primarily signals through a receptor complex consisting of *BMPR2* (type II receptor) and *ALK6*/*BMPR1B* (type I receptor), while *GDF9* utilizes *TGFβR2* and *ALK5* receptors [114,115,116]. When these ligands bind their respective receptors on granulosa cells surrounding the oocyte, they activate parallel but interconnected intracellular signaling cascades. *BMP15* binding triggers phosphorylation of receptor-regulated *SMAD 1*, *5*, and *8*, whereas *GDF9* activates *SMAD 2* and *3* [109]. These phosphorylated R-*SMADs* then form complexes with the common mediator *SMAD4*, which enables nuclear translocation and the subsequent regulation of target gene expression [117]. This bidirectional communication between oocyte and granulosa cells establishes a local regulatory environment that controls multiple aspects of follicular development, including granulosa cell proliferation, the prevention of premature luteinization, cumulus expansion, and steroidogenesis. The system is further regulated by inhibitory *SMAD*s (*SMAD6* and *SMAD7*), which provide negative feedback by interfering with R-*SMAD* phosphorylation or competing for *SMAD4* binding, thus maintaining signaling homeostasis and preventing excessive pathway activation. This follicular signaling network integrates with the broader hypothalamic–pituitary–gonadal axis through several intermediaries. Granulosa cells produce inhibins (*INHA*/*INHB*), which act as endocrine signals to suppress *FSH* secretion from the pituitary, creating a negative feedback loop that normally limits follicular development [100,118,119]. They also produce *AMH*, which inhibits primordial follicle recruitment and reduces follicular sensitivity to *FSH*, adding another regulatory layer for follicle selection [120].

*FSH* released from the pituitary binds to receptors on granulosa cells, stimulating follicular growth and estradiol production, with the sensitivity to *FSH* being modulated by *BMP15*/*GDF9* signaling. The remarkable impact of naturally occurring mutations in these pathway components illuminates their functional roles in determining litter size. Various *BMP15* mutations (such as *FecXI*, *FecXH*, *FecXB*, *FecXG*) reduce *BMP15* bioavailability or create partially functional proteins, demonstrating a precise dose-dependence where heterozygotes show increased ovulation while homozygotes are often infertile. The *BMPR1B* c.746A>G mutation, also known as the FecB (Fecundity Booroola) mutation, demonstrates a classic example of gene dosage effects where heterozygous carriers benefit from increased fertility while homozygous carriers can experience fertility problems or sterility. The *BMPR1B* c.746A>G mutation is a missense mutation that causes an amino acid substitution from glutamine to arginine at position 249 (Q249R) in the *BMPR1B* protein [39,68]. This mutation was originally identified in Booroola Merino sheep and has since been found in several sheep breeds including Hu, Ujimqin, and small-tailed Han sheep [68]. In heterozygous animals (carrying one mutant and one normal copy), the *FecB* mutation in *BMPR1B* (Q249R) increases receptor sensitivity to ligands, enhancing *SMAD1*/*5*/*8* phosphorylation at lower ligand concentrations and making granulosa cells responsive to *FSH* at smaller follicle sizes [121,122]. The altered proteins significantly enhance the sensitivity of ovarian granulosa cells to follicle-stimulating hormone, consequently resulting in increased follicular development [123]. Furthermore, this mutation demonstrates an additive effect on both ovulation rate and litter size [124]. Specifically, heterozygous animals typically exhibit four to five ovulations, which represents a substantial increase compared to the two or fewer ovulations observed in wild-type animals [123]. Mechanistically, this partial reduction in normal *BMPR1B* function appears to attenuate the inhibitory signals that ordinarily limit ovulation, thereby permitting a greater number of follicles to reach maturation and subsequently ovulate. The reproductive pathology observed in homozygous animals primarily results from the presence of two copies of the mutated gene, which consequently leads to the severe disruption of normal reproductive physiology. Specifically, ewes homozygous for mutations in *BMPR1B* demonstrate an inability to produce sufficient quantities of biologically active protein necessary for stimulating granulosa cell proliferation, thereby preventing normal follicular development [125]. Furthermore, *BMPR1B* deficiency significantly impairs the proliferation of cumulus granulosa cells while simultaneously reducing aromatase content, ultimately resulting in irregular estrous cycles [70]. Although homozygous mutants characteristically exhibit more than five ovulations [123], this apparent hyperovulation paradoxically proves detrimental to reproductive success. Rather than enhancing fertility, excessive ovulation leads to the production of poor-quality oocytes and subsequent reproductive failure. The underlying mechanism involves *BMPR1B*’s integral role within the TGF-β signaling pathway, where it functions cooperatively with downstream effectors, such as *SMAD4* [126]. Importantly, the mutation affects this critical pathway in a dose-dependent manner, creating a biphasic response; heterozygous mutations partially reduce inhibitory signaling, thereby achieving optimal ovulation enhancement, whereas homozygous mutations severely disrupt essential signaling pathways required for normal follicle development and oocyte maturation. This dose-dependent mechanism provides a molecular explanation for the paradoxical observation that some Hu sheep carrying homozygous FecB alleles continue to produce single offspring [127]. The severe disruption of normal reproductive signaling cascades in homozygous animals can ultimately result in subfertility or complete sterility, demonstrating that optimal reproductive performance requires a delicate balance of signaling pathway activity rather than complete pathway inhibition. Similarly, *GDF9* mutations like *FecGH* alter protein structure or processing, modifying *SMAD2*/*3* signaling dynamics and affecting cumulus expansion and follicular maturation [109]. These mutations do not simply eliminate function but subtly alter the signaling dynamics throughout the entire regulatory network.

The molecular consequences of these pathway alterations lead to increased litter size through several interconnected mechanisms, as follows mutations typically decrease the amount of *FSH* required for follicular development, allowing more follicles to continue development under normal *FSH* conditions; changes in granulosa cell differentiation affect inhibin production, leading to higher *FSH* secretion; normal dominance mechanisms are disrupted, allowing multiple follicles to continue development instead of undergoing atresia; altered *SMAD* signaling modifies steroid enzyme expression, changing estradiol/progesterone ratios; and anti-apoptotic signaling in granulosa cells may be enhanced, allowing more follicles to reach ovulatory stages. The entire system functions as an integrated network where oocytes communicate with granulosa cells via *BMP15*/*GDF9*, granulosa cells signal to the pituitary via inhibins, the pituitary regulates follicular development via *FSH*, and *SMAD* proteins integrate these signals intracellularly. Mutations at different points create distinct but related phenotypes, with some combinations showing additive or even multiplicative effects on ovulation rate. This sophisticated molecular dialogue ultimately determines the number of follicles that reach ovulation and, consequently, the litter size in sheep and goats, making it a paradigmatic example of how genetic variations in signaling networks translate into quantifiable reproductive traits with significant economic importance in livestock production.

## 5. Conclusions

This comprehensive review highlights the crucial role of the TGF-β superfamily in regulating litter size in small ruminants. The complex molecularinteration between *BMP15* and *GDF9*, their receptors (primarily *BMPR1B*), and downstream signaling mediators (SMADs) forms a sophisticated regulatory network that controls multiple aspects of follicular development and ovulation. Natural mutations in these pathway components alter the dynamics of signaling, ultimately increasing the number of follicles that reach ovulatory maturity, thereby enhancing litter size in sheep and goats. The evidence presented demonstrates that mutations in *BMPR1B*, *BMP15*, and *GDF9* exert their effects through several interconnected mechanisms, as follows: reducing the FSH threshold required for follicular development, disrupting follicular dominance patterns, altering steroidogenic profiles, and potentially enhancing granulosa cell survival. These changes collectively promote the development of multiple follicles under hormonal conditions that would typically support only one or two dominant follicles in non-prolific breeds. The observed gene dosage effects—where heterozygous mutations often increase ovulation while homozygous mutations sometimes cause infertility—further underscore the delicate balance required in these signaling pathways for optimal reproductive function. Additionally, the expanding body of research on *INHA*, *INHB*, *AMH*, and *SMADs* reveals additional layers of regulation that modulate the primary BMP/GDF9 signaling axis. The identification of specific SNPs in these genes associated with increased litter size across different breeds provides valuable molecular markers for selection programs. Consistent findings across geographically and genetically distinct sheep and goat populations reinforce the fundamental importance of these pathways in mammalian reproductive biology.

Looking ahead, several research directions merit further exploration. First, the functional consequences of newly identified polymorphisms require biochemical and cellular characterization to elucidate their precise effects on protein function and signaling dynamics. Second, investigating potential epistatic interactions between multiple mutations could uncover synergistic effects that might be exploited in breeding programs. Third, the role of epigenetic regulation in modulating the expression of these key genes is an emerging area that may provide additional insights into reproductive variability. From a practical standpoint, the molecular markers identified in this review present substantial opportunities for implementing marker-assisted selection strategies within small ruminant breeding programs. However, successful implementation requires careful consideration of breed-specific genetic effects and potential antagonistic relationships with other economically significant traits.

Further validation of these candidate genes across diverse sheep and goat breeds is strongly recommended, as the current literature suggests that research in this field exhibits considerable geographic and breed-specific limitations. This validation gap represents a critical barrier to the broader application of these molecular markers in global small ruminant improvement programs. Comprehensive multi-breed studies would enhance the robustness and transferability of these findings, ultimately facilitating the more effective implementation of genomic selection strategies across varied production systems and genetic backgrounds. Integrating these genetic insights with optimal nutritional and management practices will likely lead to the most substantial improvements in reproductive performance. The TGF-β superfamily stands as a master regulator of female litter size in small ruminants, with specific genetic variants providing the molecular foundation for prolificacy. Understanding these intricate signaling networks not only advances reproductive biology but also provides practical tools for improving livestock productivity and sustainability in global agricultural systems.

## Figures and Tables

**Table 1:** Summary of determinant genes (*BMPs* and *GDF9*) and their polymorphisms associated with litter size in sheep.

Genes	Polymorphism	Female Reproductive Traits	Breeds	Country	References
*BMP15* *GDF9*	c.31_33CTT	Litter size	Hu Sheep	China	[36]
*GDF9* *BMP15*	S395F and S427R	Increase the ovulation rate and litter size	Sheep	China	[37]
*BMPR1B* *BMP15* *GDF9*	c.746A>G c.31_33CTTinsdel c.994A>G	Litter size	Ujimqin, Dorper × Ujimqin crossbred Ujimqin Suffolk × Ujimqin crossbred	China	[39]
*BMP15*	g.50985975 G>A and c.755 T>C g.50988478C>A and g.50987863G>A	Litter size	Mongolia sheep Ujimqin sheep	China	[40]
*BMPRIB*	g.29362047T > C, g.29427689G > A	Litter size	Hu sheep	China	[41]
*BMP15* *GDF9*		Litter size	Akkaraman	Turkey	[42]
*BMPRIB*	p.Q249R	Litter size	Australian white, small-tailed Han, Guiqian semi-fine wool sheep	China	[43]
*BMPR1B*, *BMP15*, *GDF9*		Prolificacy	Han, Hu, Wadi, Tan sheep	China	[44]
*BMPR1B*, *BMP2*		Litter size	Chinese indigenous sheep	China	[45]
*BMPR1B*	c.687G>A g.30058882_30058873GCAGATTAAAIndel	Litter size	Gobi short-tailed sheep	China	[46]
*GDF9*		Litter size	Sudanese desert sheep	China	[48]
*BMPR1B*	C864T	Litter size	Dorset, Mongolian, small-tailed Han	China	[49]
*BMP15*	g.54285159_54285161TTA indel g.54291460G>A, g.54288671C>T, g.54285159_54285161TTA indel	Litter size	Gobi short-tailed sheep Ujimqin sheep	China	[54]
*BMPR1B*	g.30050773C>T	Fecundity	Duolang sheep	China	[55]
*GDF9*		Litter size	Bulgarian sheep	Bulgaria	[56]
*BMPR1B*	g.746A>G, g.29362047T>C, g.29427689G>A, g.29382337G>A, g.29382340G>A, g.29380965A>G	Litter size	Sheep	China	[57]
*BMPR1B*	c.746A > G	Litter size	Hu, East Friesian/Hu crossbred sheep	China	[58]
*BMPRIB*	rs427897187 G>A rs403555643 A>G	Litter size	Oula sheep	China	[59]
*BMPRIB*		Litter size	Mongolian sheep	China	[60]
*BMPRIB*	A746G, T864C, A1354G	Litter size	Qinghai Tibetan sheep	China	[61]
*BMPR1B*, *BMP15*, *GDF9*		Litter size	Dağlıç sheep	China	[62]
*BMP15*, *GDF9*		Litter size	Luzhong mutton sheep	China	[63,64]
*BMP15*, *GDF9*		Ovulation rate and litter size	Olkuska sheep	Poland	[65]
*BMP15*	c.31_33del	Litter size	Finnish Landrace sheep, Finnish, Landrace × Texel-cross sheep, composite sheep	New Zealand	[66]
*BMP15*	p.L252P	Fecundity	Cele black Sheep	China	[67]
*BMPR1B*	g.29346567C>T	Litter size	Mongolia, Ujimqin sheep	China	[68]
*BMPR1B*		Litter size	Chinese Australian white sheep	China	[69]
*BMPR1B*	g.29380965A>G	Litter size	Small-tailed Han sheep	China	[70]
*GDF9*		Litter size	Hu sheep, Mongolian sheep	China	[47,71]
*BMPR1B*, *BMP15*, *GDF9*		Litter size	Rahmani, Rahmani × Barki cross	Egypt	[72]
*GDF9*	c.1111A	Litter size	Purebred Finnish Landrace sheep, Finnish Landrace × Texel-cross sheep, composite sheep	New Zealand	[73]
*BMP2* *BMP7*	g.48462350C>T g.58171856C>G and g.58171886A>C	Litter size	Small-tailed Han sheep	China	[74]
